# Unexpected Culprit: A Case Report of Pleuropulmonary Pantoea septica Infection in a Ketamine User

**DOI:** 10.7759/cureus.65597

**Published:** 2024-07-28

**Authors:** Pyae Kyaw, Nava R Sharma, Khin Soe, Yu Shia Lin, Shaurya Sharma

**Affiliations:** 1 Internal Medicine, Maimonides Medical Center, Brooklyn, USA; 2 Infectious Diseases, Maimonides Medical Center, Brooklyn, USA

**Keywords:** pleuropulmonary infection, chronic ketamine user, pantoea species, gram negative bactermia, pantoea infection

## Abstract

*Pantoea septica*, a gram-negative bacillus typically associated with opportunistic bloodstream infections in neonatal intensive care units, rarely causes pulmonary infections in immunocompetent individuals. We present a case of a 30-year-old male with multifocal cavitary pneumonia, bilateral parapneumonic effusions, and positive blood cultures for *Pantoea septica*, occurring in the setting of prior ketamine abuse. The patient presented with fever, productive cough, chest pain, and worsening dyspnea, without significant medical history or immunocompromising conditions. Diagnostic evaluation revealed elevated inflammatory markers, characteristic radiographic findings, and successful treatment with intravenous antibiotics and pleural drainage. This case highlights the diagnostic challenge posed by *Pantoea septica* in pulmonary infections and suggests a potential link between ketamine abuse and susceptibility to uncommon pathogens, warranting further investigation into its immunomodulatory effects.

## Introduction

*Pantoea* species are gram-negative bacilli with peritrichous flagella, part of the *Enterobacteriaceae* family known for opportunistic infections in healthcare settings, particularly in neonatal intensive care units causing bloodstream infections [1]. However, they are rarely identified as primary pathogens in pulmonary infections among immunocompetent individuals. *Pantoea* exhibits a diverse ecological range, thriving in various environments, including plants, animals, soil, and water, highlighting its adaptability and potential pathogenicity under specific conditions [2,3].

Clinical understanding of *Pantoea septica* infections in pulmonary settings is limited, typically affecting immunocompromised patients or those with significant environmental exposures. Pulmonary infections caused by *Pantoea septica* present diagnostic challenges due to their unusual presentation and resemblance to more common pathogens [1]. This case describes a young male presenting with multifocal cavitary pneumonia and bilateral parapneumonic effusions, with blood cultures positive for *Pantoea septica*. His history of ketamine abuse prompted an investigation into the potential immunomodulatory effects of ketamine on pulmonary defenses and its role in predisposing individuals to infections by uncommon pathogens like *Pantoea septica*. Clarifying the relationship between ketamine abuse and susceptibility to gram-negative infections, including *Pantoea septica*, is crucial for managing similar clinical presentations and warrants further investigation into host immune responses and bacterial pathogenesis in such cases.

## Case presentation

A 30-year-old male presented to the emergency department with a one-week history of fever, productive cough, chest pain, and worsening dyspnea. Upon initial examination, he appeared acutely ill with a temperature of 101.3°F, heart rate of 94 beats per minute, respiratory rate of 18 per minute, and blood pressure of 112/66 mmHg. He denied any significant medical history, recent sick contacts, or exposure to pets, and reported working as a sushi chef.

**Figure 1:**
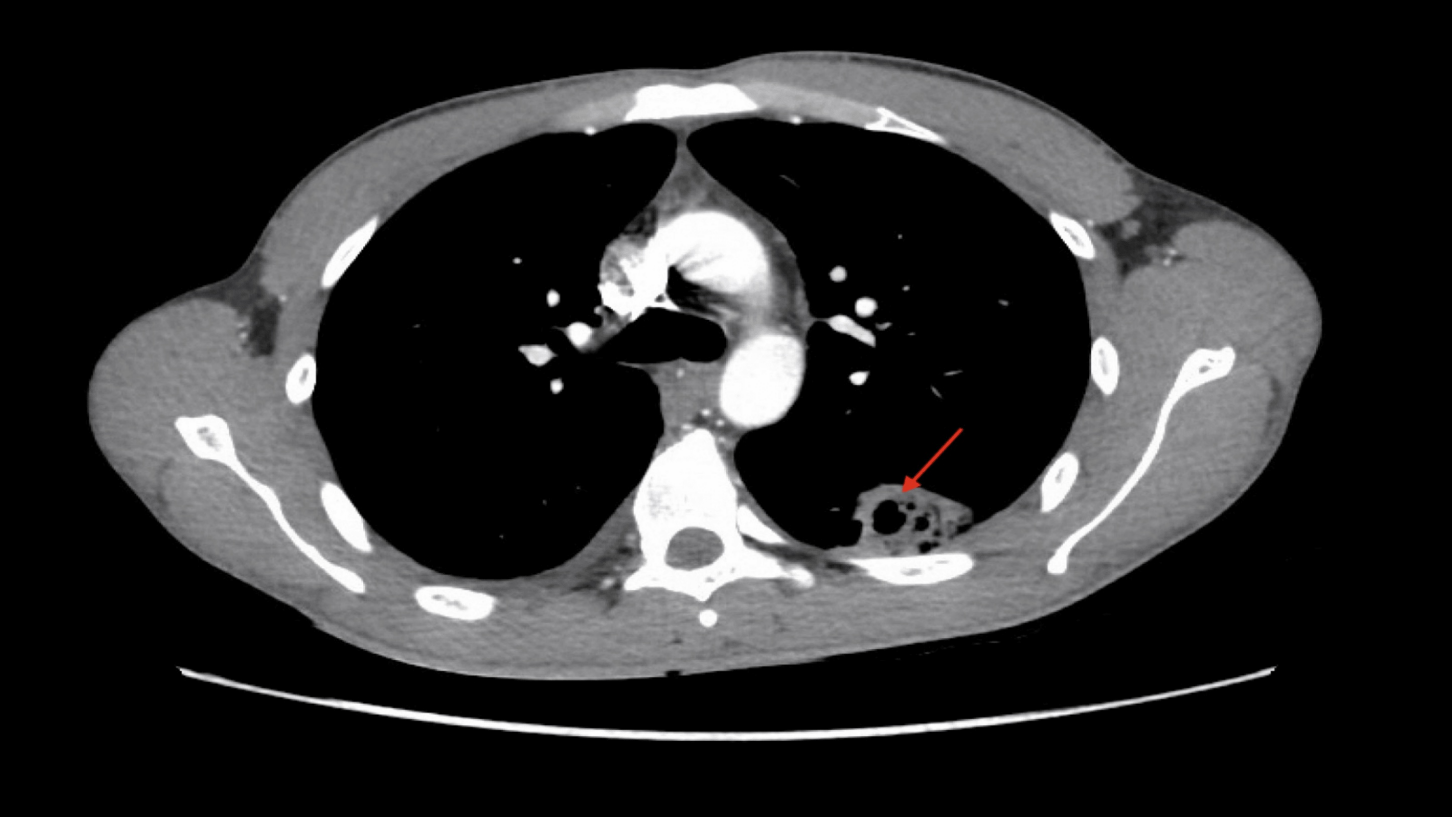
Red arrow showing cavitary lesion in the lung base. The CT scan revealed multiple discrete cavitating lesions (as shown by the red arrow) in both lungs, accompanied by several small non-cavitating lesions. Additionally, there were moderate-sized pleural effusions bilaterally, with multiple loculations within the pleural effusions.

**Figure 2:**
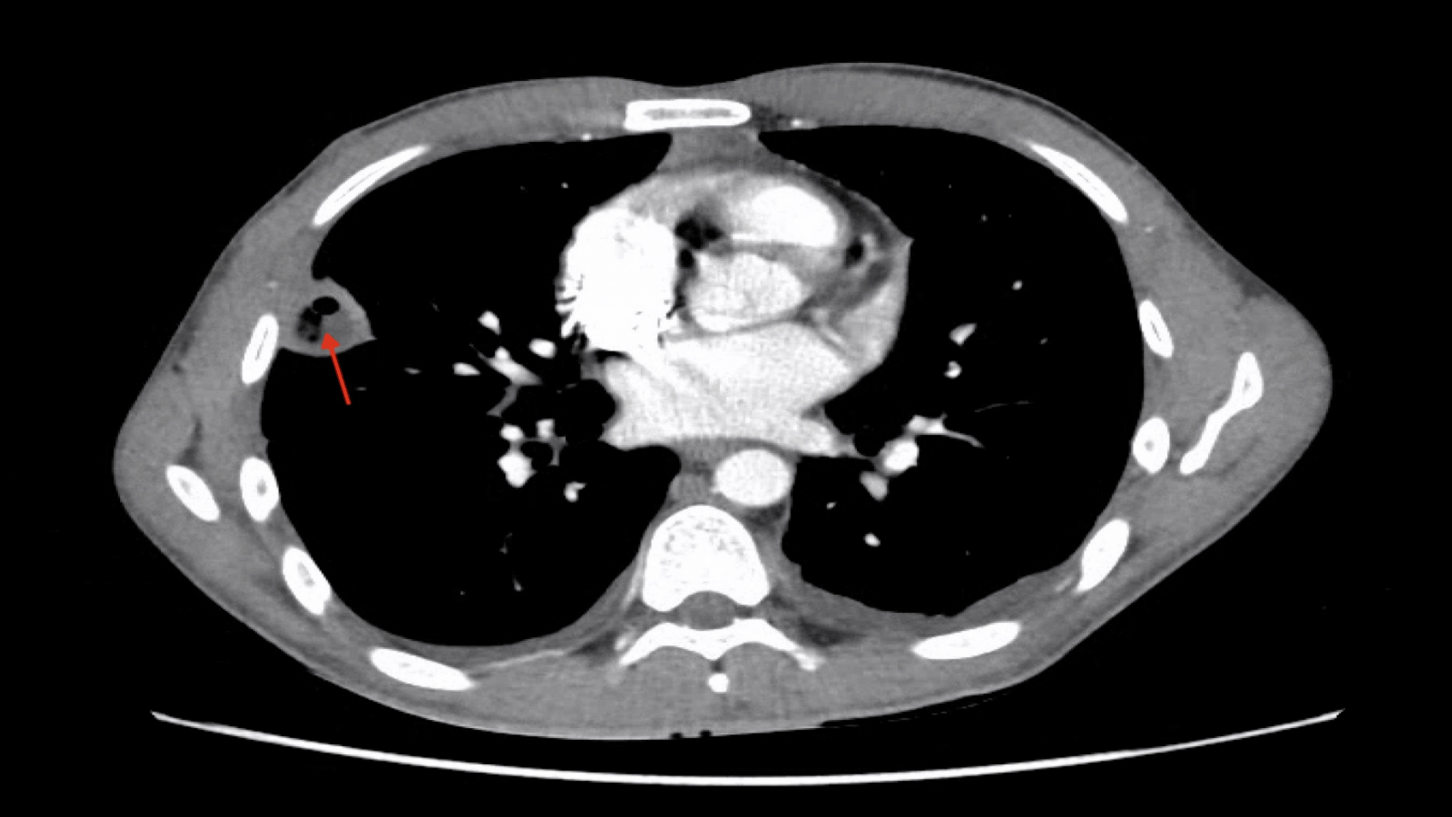
Red arrow showing cavitary lesion. A red arrow indicates a cavitary lesion located adjacent to the pleura. This lesion is accompanied by several small non-cavitating lesions along the peripheral surface adjacent to the pleura.

Laboratory investigations revealed a markedly elevated white blood cell count of 32,000/μL, predominantly neutrophilic (86%) with 3% band cells. His hemoglobin level was 12.6 g/dL, and his platelet count was 332,000/μL. Further diagnostic workup, including a comprehensive metabolic panel, legionella antigen testing, fungal infection markers, and HIV testing, returned negative results. A chest computed tomography (CT) scan demonstrated bilateral pleural effusions and cavitary lesions consistent with pneumonia and lung abscesses, as shown in Figures 1, 2.

**Figure 3:**
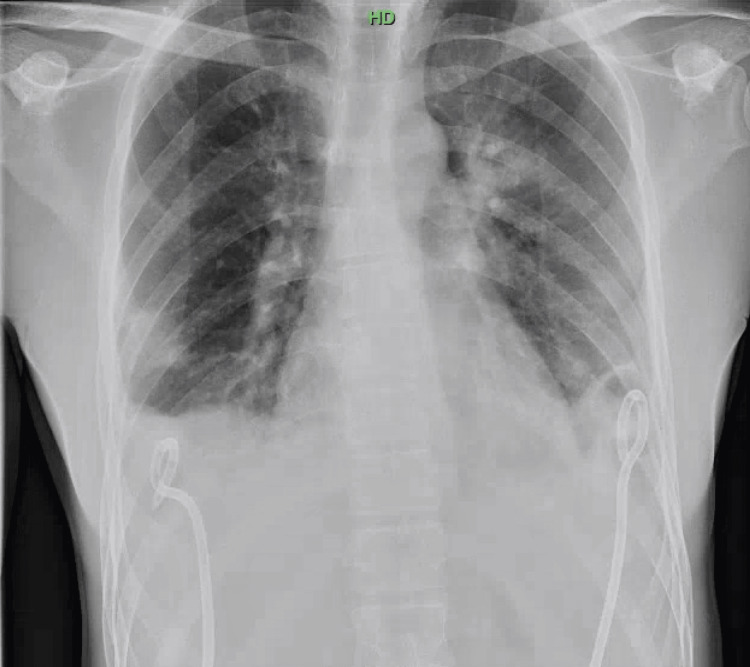
Bilateral chest tube. The image demonstrates the interval placement of bilateral chest pigtail catheters for the management of pleural effusions. There is no evidence of pneumothorax.

Blood cultures collected on admission showed the growth of a gram-negative organism by day two, later identified as *Pantoea septica*. Transthoracic echocardiography did not reveal any valvular abnormalities or vegetations, ruling out infective endocarditis. Due to the presence of bilateral pleural effusions, interventional radiology was consulted for drainage, and bilateral chest tubes were inserted, as shown in Figure 3. Analysis of the pleural fluid revealed an exudative effusion with a normal adenosine deaminase level. Cultures of pleural fluid for bacterial, mycobacterial, and fungal pathogens yielded no growth, and cytological examination did not detect malignant cells. The pleural fluid analysis result is shown in Table 1.

**Table 1:** Table showing pleural fluid analysis.

Test	Result	Normal range
Adenosine deaminase	15 U/L	<15
Albumin, fluid	2.1 g/dL	2.1 - 2.5
Amylase, fluid	20 U/L	16 and higher
Glucose, fluid	56 mg/dL	56 - 103
Lactate dehydrogenase, fluid	1336 U/L	144 and higher
Specific gravity, fluid	1.015	1.015 - 1.020
Total protein, fluid	4.1 g/dL	4.1 - 5.0
Fluid color	Yellow	Yellow
Fluid appearance	Cloudy	Cloudy
Fluid WBC	2354/mm³	-
Fluid RBC	14000/mm³	-
Fluid neutrophil	93%	-
Fluid lymphocyte	7%	-

Initial antibiotic therapy comprised intravenous cefepime and metronidazole, which was later switched to intravenous ceftriaxone upon identification of *Pantoea septica* in blood cultures, targeting the bacteremia. The patient responded well to treatment, evidenced by minimal chest tube output and clinical improvement. He was discharged on hospital day 12 with a course of oral amoxicillin-clavulanic acid and scheduled for follow-up in the infectious disease clinic.

This case underscores the diagnostic challenge posed by *Pantoea septica* as a rare pathogen causing severe pleuropulmonary infections, particularly in the absence of typical risk factors or immunocompromised states. The management highlights the importance of timely intervention with appropriate antibiotic therapy and therapeutic drainage in achieving clinical resolution.

## Discussion

*Pantoea septica*, a gram-negative bacterium characterized by its motile, unencapsulated morphology and peritrichous flagella, typically manifests as an opportunistic pathogen, primarily associated with bloodstream infections in neonatal intensive care units and rarely implicated in pulmonary infections among immunocompetent individuals [1]. The presented case underscores a unique instance of *Pantoea septica* causing multifocal cavitary pneumonia and bilateral parapneumonic effusions in a 30-year-old male with a history of ketamine abuse. This association raises intriguing questions regarding the pathogenesis of *Pantoea septica* in the pulmonary system, particularly in the context of immunocompetence and environmental exposures such as recreational drug use [2-4].

Clinical manifestations in our patient included a one-week history of fever, productive cough, and worsening dyspnea, alongside significant laboratory findings of elevated neutrophil count and radiographic evidence of pleural effusions and cavitary lesions on chest CT. Diagnosis relied heavily on blood cultures that yielded *Pantoea septica*, corroborated by pleural fluid analysis showing exudative effusion without evidence of alternative pathogens [4,5]. Notably, the patient’s occupation as a sushi chef and the history of ketamine use provided contextual clues, suggesting potential occupational or recreational exposures predisposing to this uncommon pulmonary infection.

Although no literature directly links ketamine use to *Pantoea septica* pneumonia, studies suggest that ketamine can increase susceptibility to gram-negative infections. Evidence indicates that long-term ketamine use may lead to an immunocompromised state [6-8].

The role of ketamine in modulating immune responses, particularly its reported suppression of pro-inflammatory cytokine production and nuclear factor kappa B (NF-kB) activity in pulmonary cells, merits consideration in this case [7]. In our patient's case, prolonged ketamine use likely contributed to a weakened immune system, predisposing them to *Pantoea* infection [6,7]. However, the precise mechanisms behind this association are not well-documented in the literature. To better understand the relationship between ketamine abuse and increased infection vulnerability, further research is essential [9,10].

Numerous reports of *Pantoea* infections have included susceptibility tests, showing that the bacteria are often sensitive to antibiotics such as amikacin, gentamicin, meropenem, ciprofloxacin, levofloxacin, amoxicillin/clavulanate, and broad-spectrum cephalosporins like ceftazidime and cefepime [10]. Conducting susceptibility testing is highly valuable for selecting the appropriate antibiotic treatment [9].

Treatment encompassed initial empiric therapy with intravenous cefepime and metronidazole, later adjusted to ceftriaxone upon culture confirmation of *Pantoea septica* in our case. Interventional radiology facilitated drainage of pleural effusions through bilateral chest tubes, leading to clinical improvement and eventual discharge with oral antibiotics. This case highlights the importance of prompt diagnosis, multidisciplinary management, and targeted antimicrobial therapy in achieving favorable outcomes in severe pleuropulmonary infections caused by uncommon pathogens like *Pantoea septica*.

In conclusion, while rare, infections caused by *Pantoea septica* should be considered in the differential diagnosis of severe pulmonary presentations, particularly in individuals with unique occupational or recreational exposures.

## Conclusions

Severe pulmonary conditions should raise suspicion for *Pantoea* species infections, especially among individuals with specific occupational or recreational exposures, highlighting the need for inclusion in differential diagnoses. The association with ketamine use underscores broader public health implications, urging heightened awareness and further investigation into the immunological consequences of recreational drug abuse on infectious disease susceptibility. This case prompts ongoing research efforts aimed at elucidating preventive measures and therapeutic strategies tailored to mitigate such risks in vulnerable populations.
